# Effectiveness of the serious game ‘You & I’ in changing mentalizing abilities of adults with mild to borderline intellectual disabilities: a parallel superiority randomized controlled trial

**DOI:** 10.1186/s13063-019-3608-9

**Published:** 2019-08-14

**Authors:** Suzanne Derks, Suze van Wijngaarden, Mirjam Wouda, Carlo Schuengel, Paula S. Sterkenburg

**Affiliations:** 1 Department of Clinical Child and Family Studies, Amsterdam Public Health, Vrije Universiteit Amsterdam, Van der Boechorstraat 7, 1081 BT Amsterdam, the Netherlands; 2 ASVZ, Touwbaan 1, 3360 AC Sliedrecht, the Netherlands; 3 Ons Tweede Thuis, Veteranenlaan 7, 1183 DL Amstelveen, the Netherlands; 4 Bartiméus, Oude Arnhemse Bovenweg 3, 3941 XM Doorn, the Netherlands

**Keywords:** Mentalization, Stress regulation, Intellectual disability, Serious game

## Abstract

**Background:**

Persons with mild to borderline intellectual disabilities generally show dysfunctions in mentalization and stress regulation, resulting in problematic social relationships and personal distress. Intervention programs may improve mentalizing abilities. The aim of this study is to examine the effectiveness of the serious game ‘You & I’ in changing mentalizing abilities and stress regulation in adults with mild to borderline intellectual disabilities.

**Methods:**

A two-arm, parallel, superiority randomized controlled trial will be used with 172 adults with mild to borderline intellectual disabilities. Participants will be randomly assigned to either the experimental group to play the serious game ‘You & I’ or a waitlist control group. Participants will be assessed at baseline, post intervention (5 weeks after baseline), and follow-up (6–8 weeks after post intervention). They also will fill in questionnaires for personal factors, personal development, personal well-being, social validity, autism spectrum quotient (demographic variables), mentalizing abilities (primary outcome measure), and stress regulation (secondary outcome measure).

**Discussion:**

The serious game ‘You & I’ aims to improve mentalizing abilities in adults with mild to borderline intellectual disabilities, which is expected to lead to improved regulation of stress in social relationships. The study’s unique feature is the use of a serious game to improve mentalizing abilities. If the intervention is effective, the serious game can be implemented on a broad scale in Dutch care organizations for people with intellectual disabilities as an effective preventive tool to improve mentalizing abilities.

**Trial registration:**

Netherlands Trial Register, NTR7418. Registered on 2 August 2018.

**Electronic supplementary material:**

The online version of this article (10.1186/s13063-019-3608-9) contains supplementary material, which is available to authorized users.

## Background

“Seeing oneself from the outside and others from the inside” [1]. This phrase is commonly used to describe mentalization, a concept that has been gaining attention since Fonagy reintroduced it in the early 1990s. Mentalization refers to “the mental process by which an individual implicitly and explicitly interprets the actions of himself or herself and others as meaningful on the basis of intentional mental states such as personal desires, needs, feelings, beliefs, and reasons” [2]. The capacity for mentalization is acquired in early childhood and is related to development in the domains of cognition, language, and social–emotional functioning [3]. Developmental delays in these domains in persons with mild to borderline intellectual disability (MBID) might be linked to limited capacity to mentalize [3]. It is important to examine whether interventions can help improve the mentalizing capacity of people with MBID.

Internal mental states drive how humans respond and act in certain situations. Mentalization involves recognition of mental states of the self and others, bringing beliefs, desires, intentions, goals, and emotions into awareness [4, 5]. Being aware of these mental states in the self and others facilitates coping, perceiving others as potential sources of support, and social alignment. Persons with MBID have a limited capacity to mentalize [3]. Predicting behavior of others and anticipating is therefore challenging [6–8]. Dysfunctions in mentalizing abilities may explain problems in social functioning among persons with MBID, such as destructive social interactions and social exploitation [9, 10]. Furthermore, because of limited social information processing as an executive function, people with MBID perceive more negative information, along with more difficulty remembering and processing information, limited working memory, and fewer problem-solving skills. The resulting stress makes learning mentalizing abilities more difficult [11, 12].

Within the construct of mentalization, three dimensions can be identified [13]. The first dimension is related to two modes of functioning, namely implicit and explicit functioning. Implicit mentalization refers to the unconscious, procedural, or automatic system of understanding others. Explicit mentalization involves a deliberate and conscious focus on mental content [14]. The second dimension is related to two objects, specifically the self and others. An individual can mentalize not only one’s own mental states but also the mental states of others; moreover, a person can mentalize one’s own relationships with other persons and, correspondingly, other persons’ relationships with one another [15]. The third dimension of the mentalization concept relates to its cognitive and affective aspects. Mental states in oneself and others can be cognitively focused as well as affectively laden to varying degrees [13].

According to Allen et al. [14] and Fonagy [16], humans first acquire the capacity for mentalization in the context of early attachment relationships. More specifically, the child develops mentalizing abilities within a secure attachment relationship, when the caregiver accurately and contingently mirrors the internal states of the child [13, 17]. The mirroring also needs to be *marked*. The caregiver contingently mirrors the internal state of the child [18]. This mirroring process enables the child to understand their own mental states and develop a stable sense of identity and, in turn, regulate their affect and distress [13, 18]. By also naming the mental state of the child, the child learns to recognize and name their mental state and those of others, which will improve their mentalizing abilities [19, 20].

Mentalization is not a static capacity but a dynamic, multifaceted ability whose development can be influenced by external factors such as stress [21, 22]. As Allen [15] points out: “Stress is the enemy of mentalization”. When high levels of stress are experienced, adequate mentalization becomes more difficult [23, 24]. To be more precise, the capacity to understand someone else’s mental states may be reduced, distorted, and less flexible when high levels of stress are experienced [22]. On the other hand, the ability to mentalize contributes to resilience against stress. In conclusion, support for mentalization may improve functioning even under circumstances that otherwise would have been experienced as stressful.

Awareness and understanding of mental states can be developed gradually by explicitly educating someone about mental states [25]. Empirical evidence suggests that mentalizing abilities can be improved with treatment programs such as mentalization-based therapy (MBT) [2]. In MBT, a therapist asks questions to strengthen the patient’s mentalizing abilities, such as ‘How do you think and feel about yourself and others?’, ‘Does this influence your behavior?’, ‘Can miscommunications in behavior and feelings lead to difficulties?’, and ‘How can you prevent and resolve those miscommunications?’ [16]. Randomized trials have shown that mentalization-based therapies improve interpersonal and social functioning [26, 27]. So far, treatment programs such as mentalization-based therapy have mainly focused on persons with personality disorders, while methods to stimulate the acquisition of mentalizing abilities for persons with MBID are still lacking. Moreover, current treatments for improving mentalizing abilities are time-consuming (treatment duration varying between 4 and 18 months), require supervision from qualified mental health professionals, and, consequently, are quite costly [28–30].

A promising and innovative method to improve mentalizing abilities of people with MBID is serious gaming. Serious games are computer applications that combine serious aspects, such as learning, with playful gaming elements [31]. Serious games have become increasingly popular over the years and results on their effectiveness are promising [32, 33]. Studies have also shown that serious games can be deployed successfully in the care for persons with intellectual disabilities when it comes to learning new skills and the development of abstract concepts [34]. Furthermore, MBT key elements can be realized in the serious game: a voice-over can, just like a therapist, instruct people with MBID indirectly (i.e. through interaction with an identifiable character) to recognize and name their mental state and those of others, which will improve their mentalizing abilities. Serious games can support learning which is particularly helpful for people with MBID, who learn by making abstract concepts concrete and receiving and processing new information step by step [3]. Moreover, serious games can be deployed at low cost and provide a unique learning environment wherein persons are allowed to practice new skills in a setting that is (un)likely to be realized in their daily lives [35, 36].

## Trial objective

The serious game ‘You & I’ (in Dutch: ‘Jij & Ik’) is a computer game developed with and for adults with MBID. The aim of the game is to improve mentalizing abilities, and stress regulation, focusing specifically on the three aforementioned dimensions of mentalization. In this study, the effectiveness of the serious game will be investigated, answering the following research questions: does the serious game ‘You & I’ improve mentalizing abilities in people with MBID; and does the serious game ‘You & I’ improve regulation of stress in adults with MBID?

## Hypotheses

The serious game ‘You & I’ is hypothesized to have a positive effect on mentalizing abilities, including the regulation of stress, in adults with MBID. The primary hypothesis is that being randomly assigned to the experimental group that plays the serious game will be associated with an improvement of the mentalizing abilities in adults with MBID as compared to being assigned to a waitlist control group. The secondary hypothesis is that being randomly assigned to the experimental group playing the serious game will be associated with an improvement of stress regulation in adults with MBID.

## Methods

**Fig. 1:**
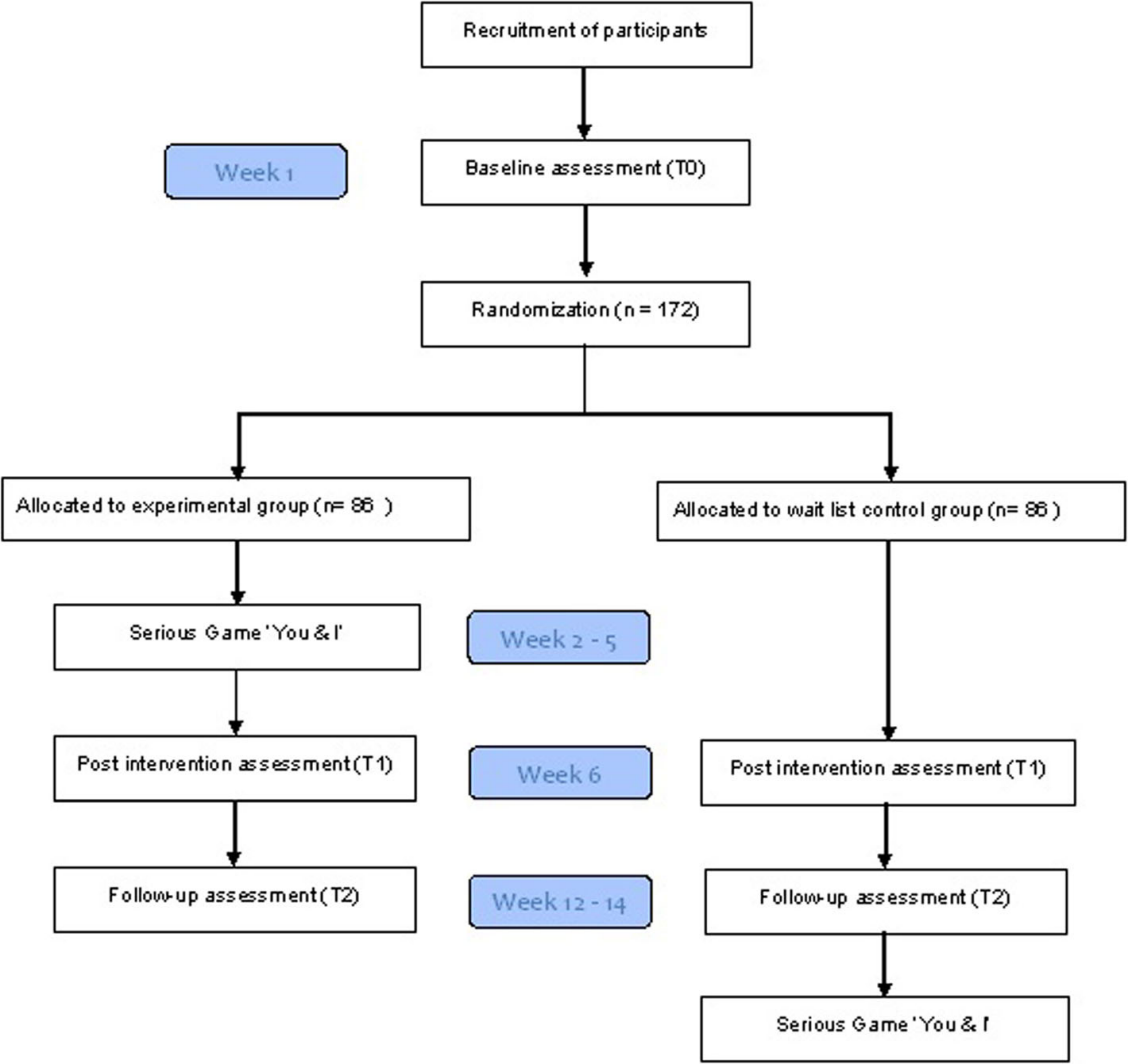
Flowchart of the study timeline

Effectiveness will be examined using a parallel superiority randomized controlled trial (RCT) with a baseline, a post test after 4 weeks, and a follow-up assessment after 6–8 weeks (see Fig. 1). The RCT includes two groups: an experimental group who play the serious game ‘You & I’; and a control group who will be placed on a waitlist. The method of this study is reported according to the Standard Protocol Items: Recommendations for Interventional Trails (see Additional file 1).

### Study population

A total of 172 adults with MBID, aged 18 years or older, will be recruited from the population of four Dutch care organizations that are specialized in disability care (Bartiméus, Ons Tweede Thuis, Cordaan, and ASVZ). Furthermore, the possibility to participate will be mentioned on websites (e.g. www.socialerelatiesenict.nl), on social media, and at various meetings. A diagnosis of MBID (IQ ranging between 50 and 85) needs to be reported, for instance, by the care organizations where the participant receives care. Inclusion criteria are having basic computer operation skills and having access to a computer with Internet connectivity. Adults need to give written consent for participation in the study and, if necessary, also their legal representative. Excluded from participation in the study are adults who are deaf and/or blind or adults who have serious mobility impairments for whom computer operation is not possible without aids.

### Sample size calculation

Linear mixed effect modeling with two conditions and three repeated measures will be conducted to analyze the effects on mentalizing abilities and stress regulation. The sample size is estimated based on previous studies measuring the mentalizing ability of perspective-taking using the Perspective Taking (PT) subscale of the Interpersonal Reactivity Index (IRI) and stress regulation using the Lifestress Inventory (LI) among people with intellectual disabilities. With the means used from both measures (for means, see [37, 38]), a desired power of 0.90, and α = 0.05, it is estimated that around 144 participants are needed, as calculated in GLIMMPSE [39]. Because there are three assessments, dropout of 20% will be taken into account. Thus, 172 participants will be recruited and randomized into two groups of approximately 86 participants in each group.

### Study procedure and randomization

Individuals who sign up for the research both via the care organization as well as via the Internet or any other route and who meet the inclusion criteria will receive an information brochure. Persons who want to participate in the study are asked to sign the consent form and return it to the researcher. In case of legal incapacitation, the legal representative of the participant is asked to sign and return a consent form on behalf of the participant.

Data collection for each participant takes 12–14 weeks. Participants will be assessed at baseline (T0), post intervention (T1, 5 weeks after baseline), and follow up (T2, 6–8 weeks after T1). During all three assessments, participants fill out a set of digital questionnaires (for all measurements, see Measures). A software program (e.g. Qualtrics or Survalyzer) will be used to gather data through the digital questionnaires. Completing the questionnaires will take up to 90 min per assessment. During all assessments, an independent researcher is present to assist the participants with completing the questionnaire at home or in their care home. The researchers will follow a standard protocol on how to assist the participants.

After informed consent and baseline assessment, blind for intervention, participants will be individually randomized into two groups using stratified randomization in combination with block randomization with varying block sizes of 4 and 6. To balance contextual factors, randomization will be stratified with regard to care organization. An independent researcher will produce the allocation schedule using a computerized random number generator and afterward conceal the schedule for the researchers. Blinding is only possible for the baseline assessment and, after that, both participants and the researchers will know to which group participants have been assigned.

After randomization, participants within the experimental group will be offered the serious game ‘You & I’, while participants within the control group will be placed on a waitlist. Participants from the experimental group will be asked to play the serious game on their own computer device at home or on a computer device of their care home. They have to complete eight gaming levels within 4 weeks, playing the game twice a week. To remind the participants to play the game, they will receive an impersonal email or text message on their phone twice a week asking them whether they have already played the game. Anonymous digital game statistics will measure the compliance of the participants (how often the computer game has been completed). After 4 weeks, the post-intervention assessment is administered, and 6–8 weeks later the participants complete the follow-up assessment. Participants from the control group can play the serious game after they have completed the follow-up assessment.

### Intervention

The intervention is a serious game called ‘You & I’ that focuses on the improvement of mentalizing abilities, including the regulation of stress. The second and last authors in collaboration with adults with MBID and healthcare professionals developed the serious game. The serious game ‘You & I’ is based on attachment theory [40], the practice-oriented book *Mentalizing in Clinical Practice* by Allen et al. [14] and the practice-oriented book *Mentalization Can Be Learned* (in Dutch: *Mentaliseren kan je leren*) by Dekker-van der Sande and Sterkenburg [3]. The participant with MBID can play the game independently on a tablet or computer.

The serious game revolves around a main character called Mo, who the player follows throughout the game by watching videos. In the first level, the player learns that Mo is sad because he misses his friend Emily, who moved to the United States. He decides to visit her and travel to the United States. The player will follow Mo on his adventure, while he leaves his house, takes the bus and the airplane, and finds his way through a foreign country to finally be able to visit Emily.

The game consists of eight gaming levels, which will take about 30–45 min to complete. The participant is asked to play the game twice a week, completing one level every time. Each level has the same structure consisting of eight different elements. That is, videos following Mo’s journey, multiple-choice questions, an emotion picture game, a stress measurer, and a game about stress. The gaming levels cover different domains of mentalization, as described by Choi-Kain and Gunderson [13]. Table 1 presents an overview of the themes and the domains of mentalization that are covered in each particular level. The first six gaming levels each cover a different domain of mentalization and levels seven and eight are so-called ‘booster levels’, implementing and repeating all domains of mentalization. By integrating the different domains of mentalization in the levels of the serious game, the player will improve their mentalizing abilities and learn how to cope with stress better.

**Table 1:** Overview of themes and domains of mentalization for each level of the serious game ‘You & I’

Level	Theme of the level	Dimensions of mentalization
1	The self	Cognitions and affections
2	Others	Cognitions and affections
3	Affective aspects	Affections, the self, and others
4	Cognitive aspects	Cognitions, the self, and others
5	Explicit functioning	Cognitions, affections, the self, and others
6	Implicit functioning	Cognitions, affections, the self, and others
7	Booster level	Cognitions, affections, the self, others, and implicit and explicit functioning
8	Booster level	Cognitions, affections, the self, others, and implicit and explicit functioning

## Measures

**Fig. 2:**
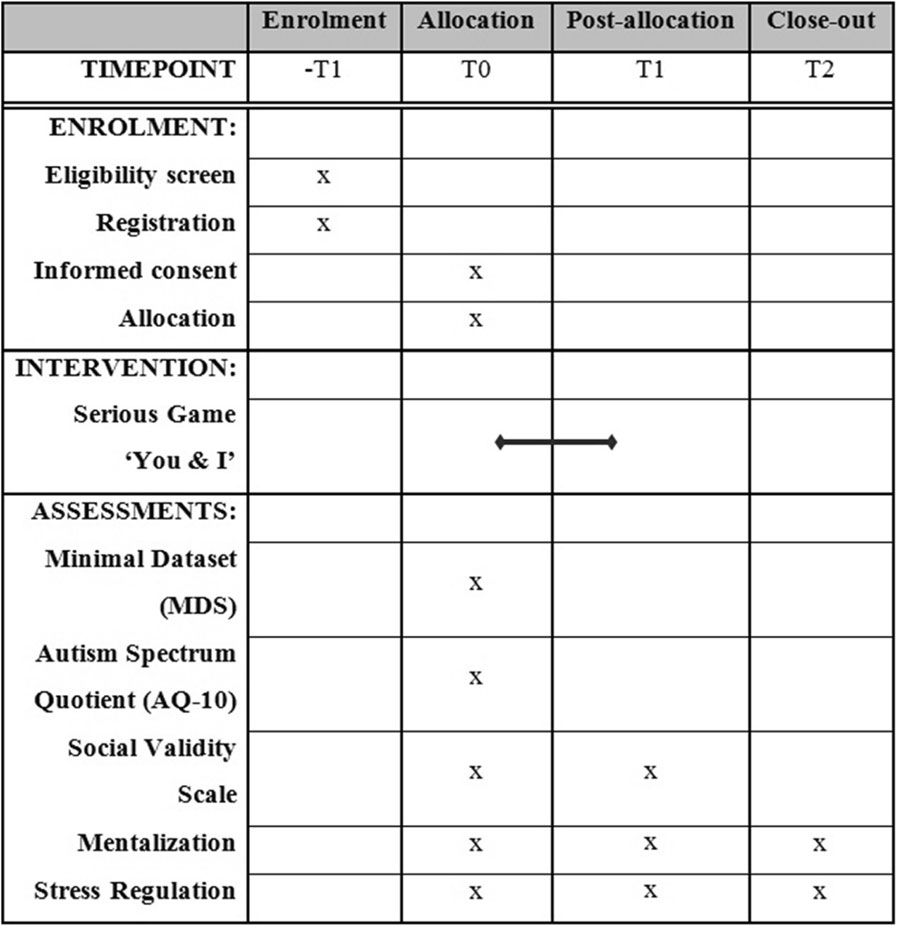
Schedule of enrollment, allocation, intervention, and assessments

All data will be collected through computerized assessments at baseline, post intervention, and follow-up assessment. Participants can fill out the digital questionnaires at home or in their care home. When needed, participants will receive support from an independent researcher, who will be present during all assessments and who will follow a standard protocol on how to assist the participants. Figure 2 provides an overview of the measures and time of assessment.

### Demographic variables

#### Minimal dataset (MDS; T0)

To measure demographic variables, the minimal dataset (MDS) ‘Basic MDS’ and ‘Basic MDS for adults with an intellectual disability’ will be used, including the Personal Wellbeing Index—Intellectual Disability (PWI-ID) [41, 42]. The MDS is a set of questions on demographic variables for everyone who collects data of persons with intellectual disabilities. The MDS focuses on questions in the following domains: personal factors, personal development, and personal well-being. The questionnaire consists of 32 items, measuring, for example, gender, age, and intellectual functioning.

#### Social Validity Scale (SVS; T0 and T1)

The SVS [43] is a questionnaire consisting of 15 questions measured on a 5-point Likert scale to assess the desirability, applicability, clarity, and efficiency of the intervention procedure. In this study, the scale will be used as described by Janssen et al. [44] and Jonker et al. [45]. During baseline assessment, participants answer questions concerning their expectations of the serious game ‘You & I’ at the post-intervention assessment questions concerning their experiences with playing the game.

#### Autism Spectrum Quotient (AQ-10; T0)

The AQ-10 [46] measures the degree to which adults with average intelligence exhibit autistic traits. The self-report questionnaire consists of 10 items measured on a 4-point Likert scale with scores ranging from *definitely agree* (1) to *definitely disagree* (4). The first items of the measure are ‘I often notice small sounds when others do not’ and ‘I usually concentrate more on the whole picture, rather than the small details’. Within a normal developing population, the AQ-10 performs well at discriminating between individuals with and without a clinical diagnosis of autism spectrum disorder [47]. The AQ-10 has not yet been used for adults with intellectual disabilities. Therefore, the items were adapted for persons with MBID. The adaptations were made by three authors (SvW, MW, PSS) and checked by collaborating researchers with MBID to align with our target group.

### Mentalization

The primary outcome measure of this study is mentalizing abilities. Several questionnaires will be used to measure mentalization, each measuring a different component of mentalization (i.e. reflective functioning, perspective-taking, emotion recognition, and the attribution of mental states). No specific effect is expected, and therefore the aggregate of the measures is tested to investigate what components are affected by the intervention.

#### The Reflective Functioning Questionnaire (RFQ; T0, T1 and T2)

The RFQ [48] is a brief self-report screening measure of mentalizing abilities. It consists of eight items measured on a 7-point Likert scale with scores ranging from *strongly disagree* (1) to *strongly agree* (7). The first three items are ‘People’s thoughts are a mystery to me’, ‘I don’t always know why I do what I do’, and ‘When I get angry, I say things without really knowing why I am saying them’. Psychometric properties are good in a normal developing population and in patients with personality disorders. For the purpose of this study, the measure was adapted for adults with intellectual disabilities by removing unnecessary wording and simplifying concepts. The adaptations were made by the first three authors and checked by collaborating researchers with MBID. Moreover, eight experimental items from the RFQ-54 were added to the questionnaire. The instrument was translated into Dutch by the second author. Then, it was translated back to English by the last author. Where necessary, adjustments were made. Any ambiguity was discussed in mail conversation with the developers of the instrument. Therefore, existing psychometric property data did not apply.

#### Radboud Faces Database (RaFD; T0, T1 and T2)

The RaFD [49] is a set of pictures depicting different emotional expressions and is used to assess emotion recognition as a part of mentalization. Participants have to view color photographs of unfamiliar faces portraying 10 different Caucasian and Moroccan adults each displaying five emotions (anger, fear, happiness, sadness, and neutral). A selection of 50 photographs has been made based on the percentage of agreement on emotion categorization, mean intensity, mean clarity, mean genuineness of the emotion, and mean valence of the photograph [49]. The pictures include averted gaze orientations (left and right) as well as direct gaze orientations (frontal). Participants have to indicate for each photograph which one of five emotions the adult depicts. The RaFD has good psychometric qualities in a normal developing population, with an average expression agreement between chosen and targeted emotions of 82% (median 88%, SD = 19%) [49].

#### Perspective Taking (PT) subscale of the Interpersonal Reactivity Index (IRI; T0, T1 and T2)

The IRI [37] is a multidimensional tool measuring interpersonal reactivity. The self-report questionnaire consists of 28 items measured on a 5-point Likert scale with scores ranging from *does not describe me well* (1) to *describes me very well* (5). The measure has four subscales, each made up of seven different items. In this study, only the PT subscale will be used. The PT subscale measures the tendency to take the psychological point of view of others. The first three items are ‘I sometimes find it difficult to see things from the other person’s point of view’, ‘I try to look at everybody’s side of a disagreement before I make a decision’, and ‘I sometimes try to understand my friends better by imagining how things look from their perspective’. The reliability of the subscale is adequate, with Cronbach’s α = 0.73 [50]. A modification of the subscale has previously been used in research on adults with moderate or mild intellectual disabilities, which also indicated adequate reliability for this population with Cronbach’s α = 0.71 [50].

#### Frith–Happé Animations Test (T0, T1 and T2)

The Frith–Happé Animations Test is added because this measure has been previously used with children with intellectual disabilities [51]. It is a nonverbal task to measure mentalizing abilities and therefore is a good addition to the other verbal questionnaires. The Frith–Happé Animations Test [51] consists of a series of computer-presented animations, each lasting 34–45 s. All animations feature one large red and one small blue triangle moving around the screen. There are three types of animations. First, Theory of Mind (ToM) animations in which it is suggested that the triangle anticipates or manipulates the ‘mental state’ of the other. Second, goal-direct action (GD) animations in which the interaction between the triangles can be described in terms of behavioral interaction. Third, random (Rd) animations in which the triangles purposelessly move around without reference to interactions, goals, or intentions.

After each animation, participants were asked ‘What was happening in the animation?’ Verbal descriptions are recorded and scored for complexity of mental state terms used (i.e. intentionality; 0–3) and accuracy of the answer given (i.e. appropriateness; 0–2). Participants are presented with two practice animations (GD and ToM) to ensure they understand the task.

### Stress regulation

The secondary outcome measure of this study is stress regulation.

#### Lifestress Inventory (LI; T0, T1 and T2)

The LI [52] is a 30-item self-report questionnaire which can be used to measure general worry, negative interpersonal interactions, and competency concerns. Participants are first asked to indicate whether they have experienced a stressor. If they do not, participants move on to the next item. If they do, they select one of four answers to indicate the impact of the stressor, ranging from *no stress* (1) to *a great deal of stress* (5). The first three items are ‘Do people treat you as though you are different?’, ‘Have you been getting on with your partner/girlfriend/boyfriend?’, and ‘Have you heard people you know arguing?’ The LI is reliable for administration of people with ID, with Cronbach’s α = 0.85 [52].

#### Perceived self-efficacy scale (stress; T0, T1 and T2)

This is a short nine-item questionnaire which can be used to measure perceived self-efficacy regarding stress regulation. The questionnaire is designed by the researchers of this study using Bandura’s guide for constructing self-efficacy scales [53] and is specifically focused on the skills that have been learned in the serious game ‘You & I’. Self-efficacy is concerned with people’s expectations of executing a particular skill, in this case stress regulation. We expect that if people are aware of actions that have the effect of regulating stress, this will lead to better stress regulation. Participants are asked on a scale from 0 (*not at all sure*) to 10 (*very sure*) how certain they are about how they can know, feel, and cope with stress. The first three items are ‘Feel in my body when I have stress’, ‘Deal with stress well’, and ‘Know that I have stress’.

## Data analysis

All statistical analysis will be conducted using SPSS version 24.0. Descriptive statistics will give insight into the characteristics of the participants. Before analyzing, outliers will be checked and, if necessary, winsorized and partial intention-to-treat analysis will be performed. Demographic variables are used to test for differences in baseline characteristics between the experimental and control groups, and are added as covariates if differences are found. For social validity, average item scores are reported.

Primary and secondary outcome measures of the study (i.e. mentalizing abilities and stress regulation) will be assessed using linear mixed-effects modeling. With Subject at the highest level and Group, Time, and the Group × Time interaction entered as fixed effects, a mixed model fits into SPSS. Furthermore, compliance will be controlled for through anonymous digital game statistics (how often the computer game is completed) and care organization will be the stratifying variable.

## Data management and monitoring

Data will be collected using online survey software. Computerized data will be stored on a secured server of Vrije Universiteit Amsterdam. The participant’s privacy is guaranteed by assigning a unique identification number to every participant. Data will be processed using these identification numbers. All researchers who will work with the research data will sign a nondisclosure agreement, stating that they will not share personal details of participants with a third party. The handling of the data will comply with the General Data Protection Regulation (GDPR). A data management plan was submitted and accepted by the funding organization of the study (ZonMw; project number 845004004). This study is also embedded in the Amsterdam Public Health (APH) Research Institute. The quality committee of APH offers a handbook to safeguard the quality of the research and performs random audits.

## Ethical considerations

Ethical approval was given by the Medical Ethics Committee of the University Medical Center Amsterdam location VUmc, the Netherlands (METc VUmc 2018.007, NL60353.029.17) and the Institutional Review Board of the Faculty of Behavioral and Movement Sciences of the Vrije Universiteit Amsterdam (VCWE-2017-171). Potential future changes to the study will be proposed to the Medical Ethics Committee as amendments, and will be described and discussed in publications of this study hereafter.

## Discussion

This article describes the study protocol of a parallel superiority randomized controlled trial for examining the effectiveness of the serious game ‘You & I’ in improving mentalizing abilities, including the regulation of stress, in adults with MBID. Participants in the experimental condition are offered to play ‘You & I’ for a duration of 4 weeks, while participants in the control condition are placed on a waitlist. It is hypothesized that participants in the experimental condition will show significant improvement in mentalizing abilities compared to participants from the waitlist control condition, as the experimental group is offered to play a serious game that is tailored for its specific purpose.

A unique feature of this study is the use of a serious game for the improvement of mentalizing abilities. This study is among the first to use a serious game specifically targeted at mentalizing abilities, particularly in adults with MBID. Serious games are promising within healthcare for persons with intellectual disabilities because their entertaining nature and familiarity to the target population offers new, alternative modalities for intervention that can improve engagement with intervention in high-risk groups. Serious games require few costs to deploy on a large scale, making these potentially cost-effective even at low levels of effectiveness. The threshold is low as it is home-based and requires only the use of a computer and Internet access [35, 54], leading to increased accessibility over face-to-face interventions such as mentalization-based therapy. If this study shows that the serious game has a positive effect on the abilities of the participants, serious games have the potential to become a standard service in healthcare for persons with MBID.

In addition to an innovative psychological intervention, the study incorporates a large group of participants from a less frequently studied population. Only little research has been carried out on adults with MBID, while they actually represent the largest group within the population of persons with intellectual disabilities [55]. A randomized controlled trial with a large sample size is especially exceptional in this field of research [56]. In this study, participants are recruited in close collaboration with four Dutch care organizations. Thus, if the serious game ‘You & I’ appears to be efficacious, the game can be instantaneously implemented as a component of already offered services for adults with MBID.

Participants who will be included in this study are recruited from a wide range of the population of adults with MBID. Persons excluded from this study (i.e. persons who are deaf and/or blind, persons who cannot operate a computer, persons younger than age 18 years) can be used to gain insight into the accessibility of this intervention for this population. Hence, participants may be diagnosed with other comorbid disorders such as autism or borderline personality disorder, have different ethnic backgrounds, come from different socioeconomic situations, and may have varying ages. This wide inclusion of participants has both advantages and disadvantages. The advantage is that external validity becomes a strong aspect of this study. The results will be easily generalizable to the population with MBID, as we expect to have a large range of participants from persons needing a lot to persons needing very little support from care organizations. Nevertheless, the disadvantage of few exclusion criteria is that the effects of this study may be smaller than expected, as the sample will be more heterogeneous.

Another limitation of this study is the use of measures that have not been specifically validated for the targeted population. As research on adults with MBID is scarce, the range of measurement instruments that are validated for this population is also more limited, especially for novel constructs such as mentalizing abilities. Therefore it is possible that the questionnaires will be influenced by language abilities of the participants [57]. To overcome this limitation, for both the primary outcome as well as the secondary outcome, both a verbal and a nonverbal measure have been selected, decreasing the chance of the results being confounded by the participants’ vocabulary competence. Furthermore, some questionnaires have been adapted to make them more suitable for the population, minimizing the possible effect of language on the results. Other researchers might use the results on the measures in future research so that measures for this population can be further developed.

In conclusion, the present study is expected to provide valuable insight into the effectiveness of the serious game ‘You & I’ for adults with MBID. If the intervention is effective, the serious game can be readily implemented on a broad scale in the care organizations for people with intellectual disabilities, thanks to the low cost of deployment. This may mean that fewer persons with MBID will suffer from problems related to mentalization deficits, such as social problems. Possibly, this may lead to less social problems and more social inclusion of persons with MBID.

## Additional file

Additional file 1: SPIRIT 2013 Checklist: Recommended items to address in a clinical trial protocol and related documents (DOC 122 kb)

## Data Availability

The dataset generated and/or analyzed during the current study will be available from the corresponding author on reasonable request.

## Abbreviations

MBID: Mild to borderline intellectual disabilities
MBT: Mentalization-based treatment
